# Supplementary material for: Patient-specific iPSC-derived photoreceptor precursor cells as a means to investigate retinitis pigmentosa
Source: eLife. 2013 Aug 27;2:e00824. doi: 10.7554/eLife.00824 (PMC3755341; doi:10.7554/eLife.00824)
Supplement: Supplementary file 1. — (A) Prioritization of exome variants. (B) Gene-specific primer sequences used for rt-PCR. F = forward primer and R= reverse primer. DOI: http://dx.doi.org/10.7554/eLife.00824.013 [file elife00824s001.docx]

**Supplementary file 1A. Prioritization of exome variants**

|  | Solid | Illumina |
| --- | --- | --- |
| All variants | 27167 | 23745 |
| Quality >50 | 16865 | 21081 |
| Coding and splice variants | 14798 | 18101 |
| dbSNP | 3046 | 3889 |
| Human exome databases | 1650 | 1937 |
| Local database | 764 | 675 |
| Family structure | 608 | 525 |

**Supplementary file 1B.** Gene specific primer sequences used for rt-PCR.

F = forward primer. R= reverse primer.

|  | |  |  | |  |
| --- | --- | --- | --- | --- | --- |
| **USH2A** **IVS40** | GCA ACA AGA GCA GCG AAT CTA C |  | **Nanog** | TTC TTC CAC CAG TCC CAA AG |  |
|  | GTA GAT TCG CTG CTC TTG TTG C |  |  | TTG CTC CAC ATT GGA AGG TT |  |
|  | |  |  | |  |
| **USH2A**  **38-F** | CAA ACC TTT GGG TGC TCA TC |  | **SOX2** | CAT CAC CCA CAG CAA ATG AC |  |
|  |  |  |  | GCA AAC TTC CTG CAA AGC TC |  |
|  | |  |  | |  |
| **USH2A**  **39-F** | ATC TGC CAC TCC AAC CAG TC |  | **c-MYC** | GCT GCT TAG ACG CTG GAT TT |  |
|  |  |  |  | AGC AGC TCG AAT TTC TTC CA |  |
|  | |  |  | |  |
| **USH2A**  **41A-R** |  |  | **KLF4** | AGA AGG ATC TCG GCC AAT TT |  |
|  | TCC TTT TGA AGT GCA GGC TT |  |  | AAG TCG CTT CAT GTG GGA GA |  |
|  | |  |  | |  |
| **USH2A**  **41C-R** |  |  | **DNMT1** | TGTACCGAGTTGGTGATGGTGTGT |  |
|  | GTC CAT GGG CTA AGA GC |  |  | TGCTGCCTTTGATGTAGTCGGAGT |  |
|  | |  |  | |  |
| **USH2A**  **41D-R** |  |  | **Pax6** | CCG GCA GAA GAT TGT AGA GC |  |
|  | GGT CCA GCA CTG TCA CCA C |  |  | GCC CGT TCA ACA TCC TTA GT |  |
|  | |  |  | |  |
|  |  |  | **Chx10** | GAG AGG GCA TTC AAC GAA GC |  |
|  |  |  |  | TCC TTG GCT GAC TTG AGG AT |  |
|  |  |  |  |  |  |
|  |  |  | **OTX2** | TTG CAC CTC CAA ACA ACC TT |  |
|  |  |  |  | GCT GTT GTT GCT GTT GTT GG |  |
|  | |  |  | |  |
|  |  |  | **CRX** | TGC GGG GAT GTG TTT CCT TC |  |
|  |  |  |  | GCC AAG GCG TTG ACA GAA TA |  |
|  | |  |  | |  |
|  |  |  | **NRL** | GCC TTC AGT CTC CTG GGA AG |  |
|  |  |  |  | GGA GGC ACT GAG CTG TAA GG |  |
|  | |  |  | |  |
|  |  |  | **Recoverin** | CTC TAC GAC GTG GAC GGT AA |  |
|  |  |  |  | CAA ACT GGA TCA GTC GCA GA |  |
|  |  |  |  |  |  |
|  |  |  | **Rhodopsin** | GGG AGA ACC ATG CCA TCA T |  |
|  |  |  |  | TCG TCT CCG TCT TGG ACA C |  |
